# Hysteretic Ion Migration and Remanent Field in Metal Halide Perovskites

**DOI:** 10.1002/advs.202001176

**Published:** 2020-08-19

**Authors:** Yongtao Liu, Nikolay Borodinov, Matthias Lorenz, Mahshid Ahmadi, Sergei V. Kalinin, Anton V. Ievlev, Olga S. Ovchinnikova

**Affiliations:** ^1^ Center for Nanophase Materials Sciences Oak Ridge National Laboratory Oak Ridge TN 37830 USA; ^2^ Joint Institute for Advanced Materials Department of Materials Science and Engineering University of Tennessee Knoxville TN 37996 USA

**Keywords:** hysteresis, ion migration, metal halide perovskites, remanent fields, spontaneous polarization

## Abstract

The gap in understanding how underlying chemical dynamics impact the functionality of metal halide perovskites (MHPs) leads to the controversy about the origin of many phenomena associated with ion migration in MHPs. In particular, the debate regarding the impact of ion migration on current–voltage (*I*–*V*) hysteresis of MHPs devices has lasted for many years, where the difficulty lies in directly uncovering the chemical dynamics, as well as identifying and separating the impact of specific ions. In this work, using a newly developed time‐resolved time‐of‐flight secondary ion mass spectrometry CH_3_NH_3_^+^ and I^−^ migrations in CH_3_NH_3_PbI_3_ are directly observed, revealing hysteretic CH_3_NH_3_^+^ and I^−^ migrations. Additionally, hysteretic CH_3_NH_3_^+^ migration is illumination‐dependent. Correlating these results with the *I*–*V* characterization, this work uncovers that CH_3_NH_3_^+^ redistribution can induce a remanent field leading to a spontaneous current in the device. It unveils that the CH_3_NH_3_^+^ migration is responsible for the illumination‐associated *I*–*V* hysteresis in MHPs. Hysteretic ion migration has not been uncovered and the contribution of any ions (e.g., CH_3_NH_3_^+^) has not been specified before. Such insightful and detailed information has up to now been missing, which is critical to improving MHPs photovoltaic performance and developing MHPs‐based memristors and synaptic devices.

## Graphical abstract

Ion migration plays critical roles in the functionalities of metal halide perovskites. Herein, using newly developed time‐resolved time‐of‐flight secondary ion mass spectrometry, hysteretic CH_3_NH_3_^+^ and I^−^ migrations in CH_3_NH_3_PbI_3_ are observed, where CH_3_NH_3_^+^ migration hysteresis is illumination‐dependent. The ion redistribution in CH_3_NH_3_PbI_3_ can lead to a permanent spontaneous current after the application of electric bias.

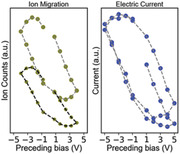

Metal halide perovskites (MHPs), as a class of outstanding optoelectronic materials,^[^ ^1^ ^]^ have attracted tremendous research interests in the past decade.^[^ ^2^ ^]^ MHPs show lots of interesting phenomena when interacting with light, such as phase segregation,^[^ ^3^, ^4^ ^]^ self‐poling,^[^ ^5^ ^]^ structural transformations,^[^ ^6^ ^]^ lattice expansion,^[^ ^7^ ^]^ giant dielectric constant,^[^ ^8^ ^]^ bulk and interfacial polarization,^[^ ^9^, ^10^ ^]^ and ultimately photovoltaic performance.^[^ ^11^ ^]^ However, intrinsic properties related to these phenomena remain elusive. Although many illumination‐dependent phenomena in MHPs are proposed to originate from the redistribution of mobile ions and defects, we are still far from fully understanding how illumination affects chemical dynamics and associated physical phenomena in MHPs because direct evidence of illumination‐dependent chemical dynamics is rare to date.

MHP is an intricate ionic system containing multiple highly mobile ionic species that can affect the material properties.^[^ ^3^, ^12^, ^13^ ^]^ For instance, both intrinsic (e.g., organic cations and halide anions)^[^ ^13^, ^14^, ^15^, ^16^, ^17^, ^18^ ^]^ and extrinsic (e.g., sodium, lithium, silver, gold, etc.)^[^ ^19^, ^20^ ^]^ mobile ions—that can annihilate defects,^[^ ^14^ ^]^ create recombination centers,^[^ ^15^, ^17^, ^21^ ^]^ or cause degradation^[^ ^16^, ^17^, ^19^ ^]^—have been discovered in MHPs. This leads to superimposed mechanisms of chemicophysical interaction in MHPs. However, ion migration in MHPs is usually investigated by indirect methods or studied in static regime, which monitor the physical responses induced by chemical changes or static chemical distribution, thus losing the precise nature of the underlying chemical dynamics. Additionally, in indirect methods, it is challenging to know which mobile species are responsible for the modification of properties because the physical responses can be induced by any ions. These result in longstanding debate about the impact of chemical dynamics on the functionalities of MHPs. A classical example is how chemical dynamics affect the current–voltage (*I*–*V*) hysteresis in MHP solar cells. Ion migration^[^ ^22^ ^]^ and associated behavior (such as band bending,^[^ ^23^ ^]^ dynamic trapping/detrapping,^[^ ^24^ ^]^ and slow transient capacitive current^[^ ^25^, ^26^ ^]^) have been proposed to interpret *I*–*V* hysteresis, as well as the effect of light‐soaking and electric poling on current–voltage hysteresis.^[^ ^27^ ^]^ Nonetheless, a definitive answer to the contribution of ion migration is still lacking. This is due to a lack in experimental evidence that directly provides information regarding ion migration and identifies the contribution of specific ions to the physical properties.

In this work, we directly observe hysteretic CH_3_NH_3_^+^ and I^−^ migrations and identify the contribution of illumination‐dependent CH_3_NH_3_^+^ migration to the *I*–*V* hysteresis observed in our lateral MHP device, which also implies the potential role of CH_3_NH_3_^+^ migration in the *I*–*V* hysteresis in other MHP‐based devices. Using an in‐house developed time‐resolved time‐of‐flight secondary ion mass spectrometry (tr‐ToF‐SIMS), we investigated the time evolution of ion distribution in MHPs during the application of external fields, such as external electric field and light illumination. The tr‐ToF‐SIMS allows us to directly observe the real‐time dynamics of any ions (such as CH_3_NH_3_^+^ and I^−^) in MHPs. Furthermore, taking advantage of machine learning approaches, i.e., nonnegative matrix factorization (NMF), we reveal that both CH_3_NH_3_^+^ and I^−^ migrations are hysteretic. The CH_3_NH_3_^+^ migration hysteresis is more illumination‐dependent, which shows larger loops under light‐on condition. In addition, using electric current measurement, we elucidate the effect of ion migration on the dynamic current behavior. We reveal that CH_3_NH_3_^+^ migration can result in a remanent field in the lateral device after the removal of electric bias, which leads to a spontaneous current under illumination. This current remains stable after 200 s of the removal of electric bias, suggesting the remanent field can last >200 s. More importantly, the plot of this spontaneous current as a function of preceding poling bias also shows a hysteresis. We expect this new understanding about the role of mobile ionic species in the hysteretic behavior of MHPs‐based device can advance MHPs photovoltaics and stimulate new research interest of MHPs functional devices, e.g., memristors and synaptic devices.

We investigated ion migration in a lateral device (Au/CH_3_NH_3_PbI_3_/Au) consisting of two gold electrodes apart from 25 µm on a surface of CH_3_NH_3_PbI_3_ thin film (device fabrication procedure is described in the Experimental Section) using an in‐house developed tr‐ToF‐SIMS technique (**Figure** **1**) that allows us to directly detect the evolution of ionic distribution at the nanoscale. Scanning electron microscopy (SEM) and X‐ray diffraction (XRD) measurements were performed to insure the quality of the CH_3_NH_3_PbI_3_ thin film. As shown in Figures S1 and S2 (Supporting Information), SEM and XRD results indicate that the film is smooth and exhibits good crystallinity, respectively. We applied a gradually increasing pulsed stepwise electric bias waveform (**Figure** **2**a) to the lateral device to induce ion migration. We named the response during application of electric biases as the on‐field response and the response in‐between bias application as the off‐field response, as indicated in Figure 2a. The off‐field response can provide information regarding not only how ions move due to electric poling but also how long the redistributed ions last. The effect of light illumination on ion migration was probed by collecting subsequent data with first light‐on followed by light‐off conditions (as shown in Figure S3, Supporting Information). A white light‐emitting diode was used for providing the light illumination, the intensity of this light‐emitting diode is characterized by a silicon solar cell, as shown in Figure S4 (Supporting Information). Figure S3b (Supporting Information) shows a time‐resolved CH_3_NH_3_^+^ distribution image obtained by tr‐ToF‐SIMS, where time evolution of CH_3_NH_3_^+^ is seen as a function of electric bias. It is seen that the CH_3_NH_3_^+^ migrates to the relatively negative electrode under the application of bias. In addition, the CH_3_NH_3_^+^ signal in the electrode region increases with time, which is due to the contamination on electrodes during measurements; nonetheless, the effect of this contamination will be excluded in the data analysis using machine learning, which will be discussed soon.

**Figure 1:**
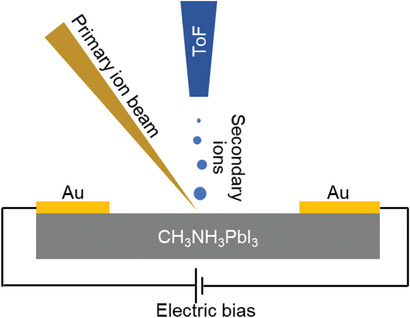
A schematic illustration of time‐resolved time‐of‐flight secondary ion mass spectrometry (tr‐ToF‐SIMS), where we continuously scan over the sample during the application of external field, such as electric field and light illumination, to obtain temporal chemical distribution.

**Figure 2:**
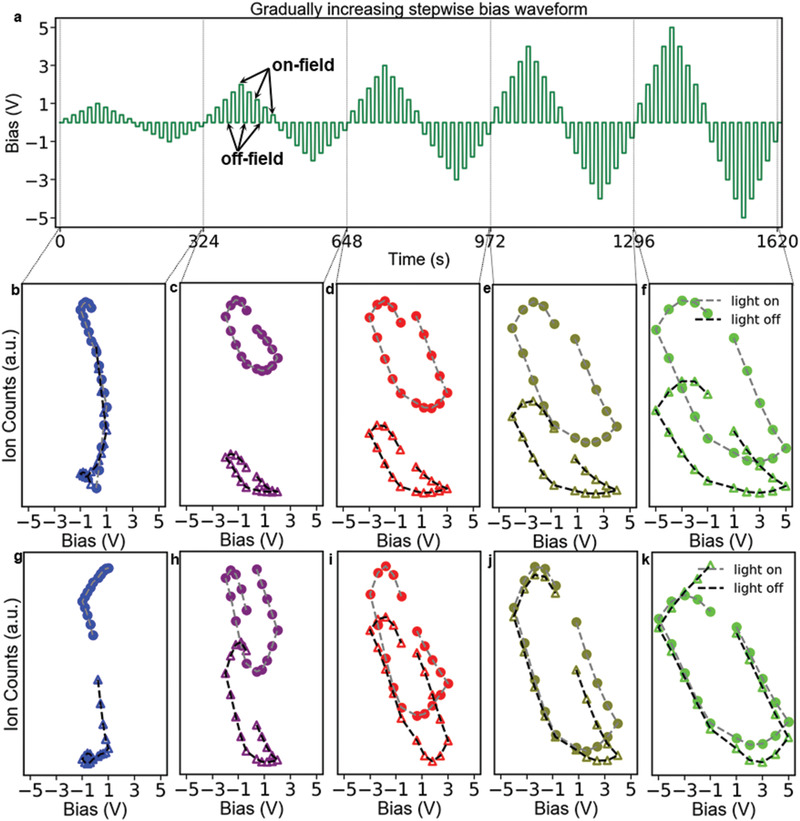
Hysteretic ion migration. a) Voltage–time (*V*–*t*) curves of the gradually increasing pulsed stepwise bias that is applied to induce ion migration; b–f) the evolution of interface CH_3_NH_3_^+^ density as a function of voltage under light‐on and light‐off conditions; g–k) the evolution of interface I^−^ density as a function of voltage under light‐on and light‐off conditions.

To gain deeper insight into the CH_3_NH_3_^+^ migration driven by electric bias, we applied machine learning analysis method of NMF to spatially isolate the tr‐ToF‐SIMS CH_3_NH_3_^+^ distribution profiles. A more detailed description of NMF analysis is in the Experimental Section. NMF allows us to separate complicated processes or signals into straightforward endmembers that are more physically interpretable. Using NMF the tr‐ToF‐SIMS result (Figure S3b, Supporting Information) was decomposed into five endmembers (Figure S5a–e, Supporting Information), each endmember describes the CH_3_NH_3_^+^ distributed in the respective regions, where we find an endmember (Figure S5c, Supporting Information) that well represents the CH_3_NH_3_^+^ distributed at Au/CH_3_NH_3_PbI_3_ interfaces (which we named as endmember *interface*). Here, we emphasize on understanding ionic behavior at Au/CH_3_NH_3_PbI_3_ interfaces as ionic behaviors at interface are critical to device performance,^[^ ^28^ ^]^ which can modulate the carriers transport barriers,^[^ ^29^, ^30^ ^]^ built‐in electric field,^[^ ^31^ ^]^ charge accumulation,^[^ ^10^, ^29^, ^32^ ^]^ etc. Nonetheless, the time evolution of each NMF endmember can be seen in Figure S5f–j (Supporting Information), which shows the periodic cycling during the application of electric bias (Figure S3a, Supporting Information) as expected. In addition, there is an endmember (Figure S5b, Supporting Information) that can describe the CH_3_NH_3_^+^ on electrodes, which we attributed to the contamination on electrodes during the measurement; as shown in Figure S5g (Supporting Information), this endmember is zero initially, suggesting no contamination on the electrodes at the beginning of the measurement, consistent with our expectation. We also tried to isolate the tr‐ToF‐SIMS result into 2–4 endmembers, but we found that five endmembers are optimal—which simultaneously separate the interested information and exclude the artifacts.

We plotted the off‐field evolution of the *interface* endmember as a function of preceding voltage (Figure S6a, Supporting Information) in order to understand how the electric poling modifies the interfacial chemistry in the Au/CH_3_NH_3_PbI_3_/Au device at ground state. Here, each step is 9 s (Figure 2a) in this measurement, so the off‐field response showing the dependence of ion distribution on preceding voltages (Figure S6, Supporting Information) suggests that CH_3_NH_3_^+^ migration can last at least 9 s after the removal of the electric bias. Detailed behaviors of the evolution of the endmember *interface* during each subperiod are shown separately in Figure 2b–f, where the hysteretic behavior is clearly seen in Figure 2c–f (no hysteretic behavior in Figure 2b). This indicates hysteretic CH_3_NH_3_^+^ migration can only happen in our device when a bipolar electric waveform with amplitude equal to or greater than 2 V (corresponding to an equivalent average electric field of 80 kV m^−1^) is applied. In addition, the CH_3_NH_3_^+^ migration hysteresis loops are similar when the bias amplitude increases from 2 to 5 V, as shown in Figure S6b,c (Supporting Information), which indicates that the normalized hysteresis loops exhibit the same shape when the bias amplitude increases from 2 to 5 V. These results (Figure S6b,c, Supporting Information) indicate that the hysteretic behavior of CH_3_NH_3_^+^ migration is independent on electric waveform amplitude and the sample degradation is negligible in this measurement (since we would see a different ion migration feature if the sample degradation is significant). However, the hysteretic behavior under light‐on and light‐off is slightly different (Figure 2b–f), seeing as higher CH_3_NH_3_^+^ signal (indicating higher CH_3_NH_3_^+^ density at Au/CH_3_NH_3_PbI_3_ interfaces) and larger hysteresis (i.e., larger loop area) under light‐on.

Iodide (I^−^) migration was also explored by tr‐ToF‐SIMS (Figure S7, Supporting Information) and NMF analyses (Figure S8, Supporting Information), where the endmember (Figure S8d, Supporting Information) representing I^−^ distributed at Au/CH_3_NH_3_PbI_3_ interface was also observed. Again, the signal (Figure S8b, Supporting Information) representing the contamination on electrodes is also excluded from the signal of interest (Figure S8d, Supporting Information) by NMF analysis. Interestingly, we also visualized hysteretic behaviors of I^−^ migration when we plotted the endmember *interface* (Figure S8d, Supporting Information) as a function of preceding voltage, as shown in Figure S9a (Supporting Information) and Figure 2g–k, suggesting I^−^ migration last at least 9 s. Similar to CH_3_NH_3_^+^, 1) the onset voltage of inducing hysteretic I^−^ migration is also 2 V (the equivalent average electric field is 80 kV m^−1^); 2) the hysteretic shape of I^−^ migration is also independent on electric waveform amplitude (Figure S9b,c, Supporting Information). In contrast, there is less difference of I^−^ migration hysteresis between light‐on and light‐off (Figure S8a, Supporting Information, and Figure 2f,g), especially when the electric bias is equal to or greater than 4 V (Figure 2j,k). This indicates that the electric field has similar effect on both CH_3_NH_3_^+^ and I^−^ migrations but the light has different effect on CH_3_NH_3_^+^ and I^−^ migration, where light condition has less effect on I^−^ migration than CH_3_NH_3_^+^ migration.

To understand the effect of ion migration on CH_3_NH_3_PbI_3_ properties, we further studied the evolution of the electric current of this lateral device by applying a pulsed stepwise electric waveform (Figure S10a, Supporting Information). The applied electric voltage (*V–t* curve) and the obtained electric current (*I–t* curve) under light‐off condition are shown in Figure S10a,b (Supporting Information), respectively. Upon the application of electric bias (on‐field), a sharp increase in electric current (Figure S10c, Supporting Information) is observed, which is followed by a decay lasting tens to hundreds of milliseconds before reaching a stable value. After the removal of electric bias (off‐field), a sharp spike in current with opposite sign is observed (Figure S10d, Supporting Information), followed by a decay that also lasts tens to hundreds of milliseconds before reaching a stable value. The measured current decay is also from tens to hundreds of milliseconds, while the ion migration (both CH_3_NH_3_^+^ and I^−^) is on the order of several seconds according to the results in Figure 2. Therefore, this current decay is unlikely due to ion migration. Instead, capacitive effect, which occurs faster than ion migration, is a more proper explanation for this current decay.^[^ ^25^ ^]^ During electric bias poling, charge accumulates at Au/CH_3_NH_3_PbI_3_ interfaces to compensate the polarization induced by the external electric field, resulting in a charging current; after removal of electric bias, the dissipation of accumulated charges induces a discharging current with opposite sign. We checked further if the current decay observed here is consistent with the capacitive effect decay by fitting them exponentially using a modified capacitive current equation

(1) $$I=I_{0}e^{-\frac{t}{RC}}+I_{1}$$

where *I*_0_ is the starting current, *RC* together specifies the rate of charge/discharge of a capacitor (which is named as *RC* time constant), where *R* is the resistance of the circuit and *C* is the capacity of the device. Considering CH_3_NH_3_PbI_3_ is a semiconductor, we also introduced a stable current *I*_1_ to represent the current due to the semiconducting nature of CH_3_NH_3_PbI_3_. As expected, both charging current and discharging current decay exponentially with time (Figure S10a–d, Supporting Information), consistent with capacitive current decay. We also obtained the RC time constant of this device under various voltages (Figure S12, Supporting Information), which increases with the amplitude of the increasing voltage. This is because that the bias poling induces a remanent field persisted after the removal of the bias (the remanent field will be discussed later); as a consequence, the device condition is slightly different at each step. Nonetheless, all the *RC* time constants are similar (in the range of 17.12–22.09 ms). It is worth noting that we expect faster decay (smaller *RC* time constant) with the bias voltage increases if this decay process is dominated by ion migration as the ions are expected to move faster under larger bias voltages; therefore, the trend of RC time constant (Figure S12, Supporting Information) also suggests this fast decay process is not dominated by ion migration.

The *I–t* curve under light‐on condition was also studied, as shown in Figure S13a (Supporting Information). Similar to that under light‐off, light‐on electric current also shows a sharp increase in electric current (Figure S13b, Supporting Information) upon the application of electric bias (on‐field), which is followed by a fast decay lasting tens to hundreds of milliseconds. After the removal of electric bias (off‐field), a sharp spike in current with opposite sign is observed (Figure S13c, Supporting Information), followed by a fast decay lasting tens to hundreds of milliseconds. These decays can also be fitted exponentially (Figure S14, Supporting Information). In addition, the *RC* time constant also increases with increasing bias voltages (Figure S15, Supporting Information). Therefore, we believe the dominant origin of these fast decays (on‐field decays and off‐field decays) is still the capacitive effect. However, when we scrutinized the on‐field current and the off‐field current under light‐on condition, we found that the on‐field current slowly increases after the initial fast decay (as shown in the inset of Figure S13b, Supporting Information) and the off‐field current slowly decreases after the initial fast decay (as shown in the inset of Figure S13c, Supporting Information).

In tr‐ToF‐SIMS ion migration studies, we observed ion migration can last several seconds, consistent with the slow behavior observed in the insets of Figure S13b,c (Supporting Information). Accordingly, we attribute the slow increase of on‐field current and the slow decrease of off‐field current to the ion migration. In addition, these slow behaviors are not observed under light‐off condition, as shown in the insets of Figure S10c,d (Supporting Information), suggesting these slow behaviors are governed by the ion whose behavior is illumination‐dependent, that is, CH_3_NH_3_^+^ ion.

Another feature we observed in *I–t* curve under light‐on condition is that the off‐field current does not reach 0 A after 9 s relaxation, as shown in the inset of Figure S13c (Supporting Information) (we refer this current to spontaneous current hereafter). This motivated us to explore whether this spontaneous current depends on the preceding bias (i.e., on‐field bias). Therefore, we only plotted the off‐field *I–t* curves under light‐on condition to highlight the correlation between the spontaneous current and the on‐field bias, as shown in **Figure** **3**a. It is clearly seen that the spontaneous current depends on the on‐field bias. In the first‐half cycle (from 0 to 162 s, in Figure 3a), where the on‐field biases are positive, the spontaneous current is negative, suggesting the positive poling generates a negative remanent field that induces a negative spontaneous current after the removal of the bias. When plotting the spontaneous current as a function of the preceding bias, we observed a hysteresis loop, as shown in Figure 3b. Similar analyses were also applied on the *I–t* curve under light‐off condition, as shown in Figure 3c,d. However, all related behaviors are much weaker, or even invisible under light‐off. In particular, the hysteresis in Figure 3d is almost closed.

**Figure 3:**
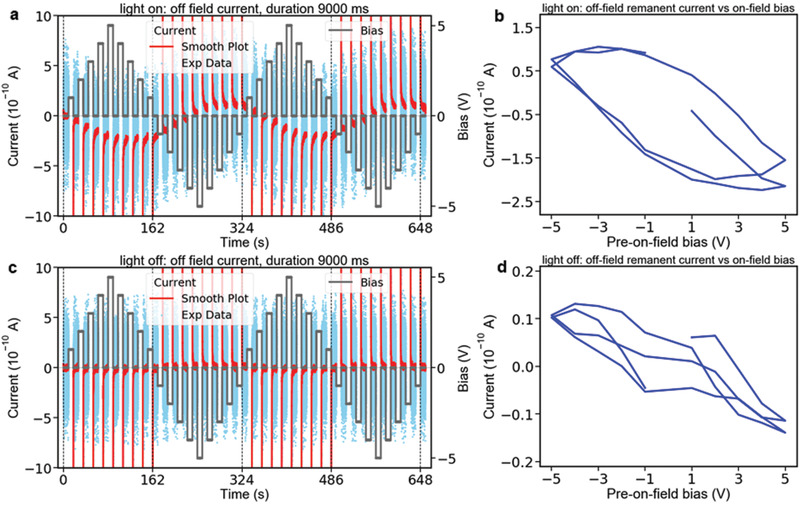
Current–time (*I*–*t*) curves after electric poling. a) The off‐field *I*–*t* curve under light‐on condition along with the *V*–*t* curve. b) The plot of the spontaneous current under light‐on condition as a function of the on‐field bias. c) The off‐field *I*–*t* curve under light‐off condition along with the *V*–*t* curve. d) The plot of the spontaneous current under light‐off condition as a function of the on‐field bias.

After revealing the hysteretic spontaneous current, we wonder if this spontaneous current is just a temporal current originating from the slow ion diffusion. Therefore, we performed an additional *I–t* measurement to examine how long the spontaneous current can last. As shown in **Figure** **4**a, we applied 5 V bias to the lateral device for 9 s as for other experiments, then left the device to relax under 0 V for longer time—200 s. The behavior of the electric current during bias poling is the same to that we observed in Figure 3a. When we zoomed in (the inset of the Figure 4a), we can still observe the current around 218 s, suggesting that the spontaneous current can last >200 s. Even if we only measured the current for 200 s after the removal of the electric field, we speculate this spontaneous current can persist permanently because it is still very stable after 200 s. We also performed this measurement by applying 3 V bias to the device, as shown in Figure 4b. The overall phenomena are similar to that by 5 V poling in Figure 4a. In addition, the spontaneous current after 3 V poling (the inset of Figure 4b) is smaller than that after 5 V poling (Figure 4a) as expected. This measurement was also performed under light‐off condition as a comparison. As shown in Figure S16 (Supporting Information), both the currents after 5 V poling (Figure S16a, Supporting Information) and 3 V poling (Figure S16b, Supporting Information) are almost zero. This is also consistent with our observation in Figure 3c.

**Figure 4:**
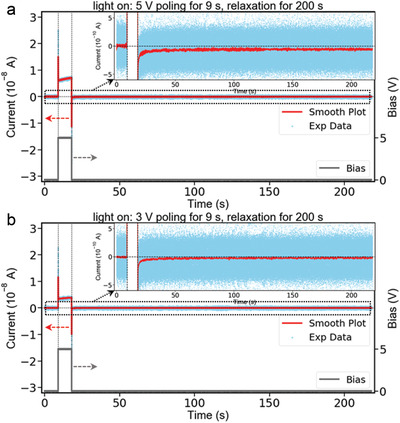
Current behavior under light‐on. a) *I*–*t* curve by 5 V poling for 9 s and relaxing for 200 s; the inset is a zoomed‐in *I*–*t* curve to show the spontaneous current behavior. b) *I*–*t* curve by 3 V poling for 9 s and relaxing for 200 s; the inset is a zoomed‐in *I*–*t* curve to show the spontaneous current behavior.

According to these observations, we propose that the CH_3_NH_3_^+^ redistribution partially persists after the removal of the bias voltage, which induces a permanent remanent field leading to a spontaneous current in the device for following reasons. From the electric current measurements, we clearly see difference of the device condition after bias poling between light‐on and light‐off. Although we attributed the initial fast decay to capacitive effect, the decay rate should depend on the device condition. The observation of hysteretic behavior suggests that the device condition is different after various bias poling; therefore, the spread of the decay rate (*RC* time constant) under various bias voltages can be an indicator of how much the device condition is modified by bias poling. As seen in Figures S12 and S15 (Supporting Information), the spread of the *RC* time constant is Δ4.97 ms (from 17.12 to 22.09 ms) under light‐off, while it is Δ6.81 ms (from 17.89 to 24.70 ms) under light‐on, suggesting that the device is modified more significantly under light‐on. I^−^ migration is the same under light‐on and light‐off (Figure 2k), which should not be responsible for these changes. In contrast, CH_3_NH_3_^+^ migration depends on light condition (Figure 2f), implying it is a reason for the device condition change. Moreover, the similarity between the spontaneous current hysteresis and CH_3_NH_3_^+^ migration hysteresis depending on light illumination—both CH_3_NH_3_^+^ migration and spontaneous current show larger hysteresis under light‐on—is a more straightforward implication for the correlation between the CH_3_NH_3_^+^ migration and the spontaneous current. Nonetheless, we would like to note that the observation in tr‐ToF‐SIMS is ionic specific, while the *I*–*V* data consist of multiple contributions including mobile ions, structural distortion, etc. Even if our results strongly suggest that CH_3_NH_3_^+^ migration contributes to the current hysteresis, we cannot exclude other contributing factors here. We think the current behavior should have an integrated and interactive origin, where CH_3_NH_3_^+^ migration plays an indispensable role.

In summary, we for the first time directly observed hysteretic CH_3_NH_3_^+^ and I^−^ migrations using an in‐house developed tr‐ToF‐SIMS technique. We found that CH_3_NH_3_^+^ migration hysteresis is more illumination‐dependent than I^−^ migration. We revealed that the bias‐induced ion migration under illumination can result in a remanent current lasting more than 200 s, while this remanent current is negligible under light‐off. Moreover, the plot of this remanent current under illumination as a function of the bias voltage shows a current–voltage hysteresis loop. Combining the hysteretic ion migration with current–voltage characterization, we revealed that CH_3_NH_3_^+^ migration contributes to the illumination‐dependent current–voltage hysteresis. According to the light‐dependent behavior of CH_3_NH_3_^+^ migration, we propose that the CH_3_NH_3_^+^ migration also play critical roles in other illumination‐dependent phenomena in MHPs. These results offer new and insightful information regarding the understanding of MHPs functionality, which will advance the development of MHPs photovoltaics and stimulate research interests of other MHPs‐based functional devices, such as memristors and synaptic devices. In addition, the demonstrated technique, tr‐ToF‐SIMS, is also useful for other material systems; we expect this method will also be helpful to investigate the chemical changes in other materials.

## Experimental Section

##### CH_3_NH_3_PbI_3_ Synthesis and Lateral Device Fabrication

The CH_3_NH_3_PbI_3_ samples were synthesized on Swiss glass slides. The glass slides were precleaned using deionized water, acetone, and isopropanol, respectively. The CH_3_NH_3_PbI_3_ was synthesized by spin‐casting the precursor (0.8 m lead (II) acetate trihydrate and 2.4 m CH_3_NH_3_I in dimethylformamide) and annealed at 100 °C for 30 min in a N_2_‐filled glove box. The gold (Au) electrodes were deposited in a clean room using an electron beam evaporator and a mask.

##### Time‐of‐Flight Secondary Ion Mass Spectrometry

ToF‐SIMS measurements were performed using ToF.SIMS5NCS instrument (IONTOF GmbH, Germany). Experiments were carried out in positive ion mode with spectra calibrated using the Na^+^, CH_3_NH_3_^+^, and Pb^+^ peaks for CH_3_NH_3_^+^ studies and in negative ion mode for I^−^ studies with spectra calibrated using the I^−^, Au^−^, and I_2_^−^ peaks. A Bi_3_^+^ analysis beam was used to scan a sample area with 128 × 128 px random pattern. Illumination condition was changed by a built‐in LED white light in ToF‐SIMS chamber. Bias voltages were applied externally using a Tektronix AFG1022 arbitrary function generator (Tektronix, Beaverton, OR) and Lab VIEW. The current‐time (*I*–*t*) curve measurements were also conducted in the ToF‐SIMS chamber by applying electric waveform using Lab VIEW.

##### Nonnegative Matrix Factorization

NMF is a classical form of the unsupervised unmixing machine learning approach, which is an algorithm in multivariate analysis where an input data is factorized into a number of endmember‐abundance pairs with the property that all endmembers have no negative elements. This method has been widely utilized in materials science in analyzing experimental data.^[^ ^33^, ^34^ ^]^ This method is particularly suitable for analyzing ToF‐SIMS data, where the nonnegativity is inherent because molecular mass cannot be negative.

In this work, NMF data analysis was performed on a desk computer using Python 3.6 and scikit‐learn 0.19.2 library, where the CH_3_NH_3_^+^ distribution profile *X_i_* is unmixed on a linear combination of endmembers *v_j_* and noise *N_i_*

(2) $$X_{i}=\sum_{j}A_{ij}v_{j}+N_{i}$$

endmembers are calculated by minimizing noise *N_i_* component, subject to *v_i_* ≥ 0, which means every endmember will be nonnegative. More information regarding NMF and its application in material science can be found elsewhere.^[^ ^33^ ^]^

##### Statistical Analysis

Experiments and data analysis details were demonstrated above in each subsection of the Experimental Section. The current–time results are shown as raw data and smoothed data, the smoothed data are obtained by smoothing the raw data with Savitzky–Golay filter in Python.

## Conflict of Interest

The authors declare no conflict of interest.

## Supporting information

Supporting Information

## References

[1] Y. Zhao , K. Zhu , Chem. Soc. Rev. 2016, 45, 655. 26645733 10.1039/c4cs00458b

[2] a) A. Kojima , K. Teshima , Y. Shirai , T. Miyasaka , J. Am. Chem. Soc. 2009, 131, 6050; 19366264 10.1021/ja809598r

[3] E. T. Hoke , D. J. Slotcavage , E. R. Dohner , A. R. Bowring , H. I. Karunadasa , M. D. McGehee , Chem. Sci. 2015, 6, 613. 28706629 10.1039/c4sc03141e PMC5491962

[4] D. J. Slotcavage , H. I. Karunadasa , M. D. McGehee , ACS Energy Lett. 2016, 1, 1199.

[5] Y. Deng , Z. Xiao , J. Huang , Adv. Energy Mater. 2015, 5, 1500721.

[6] R. Gottesman , L. Gouda , B. S. Kalanoor , E. Haltzi , S. Tirosh , E. Rosh‐Hodesh , Y. Tischler , A. Zaban , C. Quarti , E. Mosconi , J. Phys. Chem. Lett. 2015, 6, 2332. 26266613 10.1021/acs.jpclett.5b00994

[7] a) Y. Liu , A. V. Ievlev , L. Collins , N. Borodinov , A. Belianinov , J. K. Keum , M. Wang , M. Ahmadi , S. Jesse , K. Xiao , Adv. Opt. Mater. 2019, 7, 1901451;

[8] E. J. Juarez‐Perez , R. S. Sanchez , L. Badia , G. Garcia‐Belmonte , Y. S. Kang , I. Mora‐Sero , J. Bisquert , J. Phys. Chem. Lett. 2014, 5, 2390. 26279565 10.1021/jz5011169

[9] C. Zhao , B. Chen , X. Qiao , L. Luan , K. Lu , B. Hu , Adv. Energy Mater. 2015, 5, 1500279.

[10] M. Ahmadi , L. Collins , K. Higgins , D. Kim , E. Lukosi , S. V. Kalinin , ACS Appl. Mater. Interfaces 2019, 11, 41551. 31595742 10.1021/acsami.9b16287

[11] E. L. Unger , E. T. Hoke , C. D. Bailie , W. H. Nguyen , A. R. Bowring , T. Heumüller , M. G. Christoforo , M. D. McGehee , Energy Environ. Sci. 2014, 7, 3690.

[12] a) E. Mosconi , F. De Angelis , ACS Energy Lett. 2016, 1, 182; 10.1021/acsenergylett.1c00553 PMC8763376 35059501

[13] a) M. Lai , A. Obliger , D. Lu , C. S. Kley , C. G. Bischak , Q. Kong , T. Lei , L. Dou , N. S. Ginsberg , D. T. Limmer , Proc. Natl. Acad. Sci. USA 2018, 115, 11929; 30397127 10.1073/pnas.1812718115 PMC6255190

[14] D. W. DeQuilettes , W. Zhang , V. M. Burlakov , D. J. Graham , T. Leijtens , A. Osherov , V. Bulović , H. J. Snaith , D. S. Ginger , S. D. Stranks , Nat. Commun. 2016, 7, 11683. 27216703 10.1038/ncomms11683 PMC4890321

[15] C. Li , A. Guerrero , S. Huettner , J. Bisquert , Nat. Commun. 2018, 9, 5113. 30504825 10.1038/s41467-018-07571-6 PMC6269531

[16] a) S. Kim , S. Bae , S.‐W. Lee , K. Cho , K. D. Lee , H. Kim , S. Park , G. Kwon , S.‐W. Ahn , H.‐M. Lee , Sci. Rep. 2017, 7, 1200; 28446755 10.1038/s41598-017-00866-6 PMC5430925

[17] H. Yuan , E. Debroye , K. Janssen , H. Naiki , C. Steuwe , G. Lu , M. Moris , E. Orgiu , H. Uji‐i , F. De Schryver , J. Phys. Chem. Lett. 2016, 7, 561. 26804213 10.1021/acs.jpclett.5b02828 PMC4745111

[18] a) Y. Liu , A. V. Ievlev , L. Collins , A. Belianinov , J. K. Keum , M. Ahmadi , S. Jesse , S. T. Retterer , K. Xiao , J. Huang , Adv. Electron. Mater. 2020, 6, 1901235;

[19] Z. Li , C. Xiao , Y. Yang , S. P. Harvey , D. H. Kim , J. A. Christians , M. Yang , P. Schulz , S. U. Nanayakkara , C.‐S. Jiang , Energy Environ. Sci. 2017, 10, 1234.

[20] a) J. Zhang , R. Chen , Y. Wu , M. Shang , Z. Zeng , Y. Zhang , Y. Zhu , L. Han , Adv. Energy Mater. 2018, 8, 1701981;

[21] A. Pockett , M. J. Carnie , ACS Energy Lett. 2017, 2, 1683.

[22] H. J. Snaith , A. Abate , J. M. Ball , G. E. Eperon , T. Leijtens , N. K. Noel , S. D. Stranks , J. T.‐W. Wang , K. Wojciechowski , W. Zhang , J. Phys. Chem. Lett. 2014, 5, 1511. 26270088 10.1021/jz500113x

[23] a) C. Li , S. Tscheuschner , F. Paulus , P. E. Hopkinson , J. Kießling , A. Köhler , Y. Vaynzof , S. Huettner , Adv. Mater. 2016, 28, 2446; 26823239 10.1002/adma.201503832

[24] H. Yu , H. Lu , F. Xie , S. Zhou , N. Zhao , Adv. Funct. Mater. 2016, 26, 1411.

[25] B. Chen , M. Yang , X. Zheng , C. Wu , W. Li , Y. Yan , J. Bisquert , G. Garcia‐Belmonte , K. Zhu , S. Priya , J. Phys. Chem. Lett. 2015, 6, 4693. 26550850 10.1021/acs.jpclett.5b02229

[26] O. Almora , I. Zarazua , E. Mas‐Marza , I. Mora‐Sero , J. Bisquert , G. Garcia‐Belmonte , J. Phys. Chem. Lett. 2015, 6, 1645. 26263328 10.1021/acs.jpclett.5b00480

[27] M. De Bastiani , G. Dell'Erba , M. Gandini , V. D'Innocenzo , S. Neutzner , A. R. S. Kandada , G. Grancini , M. Binda , M. Prato , J. M. Ball , Adv. Energy Mater. 2016, 6, 1501453.

[28] a) I. Levine , P. K. Nayak , J. T.‐W. Wang , N. Sakai , S. Van Reenen , T. M. Brenner , S. Mukhopadhyay , H. J. Snaith , G. Hodes , D. Cahen , J. Phys. Chem. C 2016, 120, 16399;

[29] H. Zhang , C. Liang , Y. Zhao , M. Sun , H. Liu , J. Liang , D. Li , F. Zhang , Z. He , Phys. Chem. Chem. Phys. 2015, 17, 9613. 25772648 10.1039/c5cp00416k

[30] a) B. C. O'Regan , P. R. Barnes , X. Li , C. Law , E. Palomares , J. M. Marin‐Beloqui , J. Am. Chem. Soc. 2015, 137, 5087; 25785843 10.1021/jacs.5b00761

[31] a) M. Stumpp , R. Ruess , J. Horn , J. Tinz , C. Richter , D. Schlettwein , Phys. Status Solidi A 2016, 213, 38;

[32] Y. Zhao , C. Liang , H. Zhang , D. Li , D. Tian , G. Li , X. Jing , W. Zhang , W. Xiao , Q. Liu , Energy Environ. Sci. 2015, 8, 1256.

[33] a) R. Kannan , A. Ievlev , N. Laanait , M. A. Ziatdinov , R. K. Vasudevan , S. Jesse , S. V. Kalinin , Adv. Struct. Chem. Imaging 2018, 4, 6; 29755927 10.1186/s40679-018-0055-8 PMC5928180

[34] a) T. A. Doherty , A. J. Winchester , S. Macpherson , D. N. Johnstone , V. Pareek , E. M. Tennyson , S. Kosar , F. U. Kosasih , M. Anaya , M. Abdi‐Jalebi , Nature 2020, 580, 360; 32296189 10.1038/s41586-020-2184-1

