# Supplementary material for: Hysteretic Ion Migration and Remanent Field in Metal Halide Perovskites
Source: Adv Sci (Weinh). 2020 Aug 19;7(19):2001176. doi: 10.1002/advs.202001176 (PMC7539187; doi:10.1002/advs.202001176)
Supplement: Supplementary file 1 — Supporting Information [file ADVS-7-2001176-s001.pdf]

© 2020 Wiley-VCH GmbH

ADVANCED SCIENCE Open Access

Supporting Information

for *Adv. Sci.,* DOI: 10.1002/advs.202001176

Hysteretic Ion Migration and Remanent Field in Metal Halide Perovskites

*Yongtao Liu, Nikolay Borodinov, Matthias Lorenz, Mahshid Ahmadi, Sergei V. Kalinin, Anton V. Ievlev, and Olga S. Ovchinnikova**

Supporting Information:

Hysteretic Ion Migration and Remanent Field in Metal Halide Perovskites

*Yongtao Liu, Nikolay Borodinov, Matthias Lorenz, Mahshid Ahmadi, Sergei V. Kalinin, Anton V. Ievlev, Olga S. Ovchinnikova**

Mr. Y. Liu, Dr. N. Borodinov, Dr. M. Lorenz, Dr. S. V. Kalinin, Dr. A. V. Ievlev, Dr. O. S. Ovchinnikova
Center for Nanophase Materials Sciences
Oak Ridge National Laboratory
Oak Ridge, Tennessee 37830, United States
E-mail: ovchinnikovo@ornl.gov.

Mr. Y. Liu, Dr. M. Ahmadi
Joint Institute for Advanced Materials
Department of Materials Science and Engineering
University of Tennessee
Knoxville, Tennessee 37996, United States

Notice: This manuscript has been authored by UT-Battelle, LLC, under Contract No. DE-AC0500OR22725 with the U.S. Department of Energy. The publisher, by accepting the article for publication, acknowledges that the United States Government retains a non-exclusive, paid-up, irrevocable, world-wide license to publish or reproduce the published form of this manuscript, or allow others to do so, for the United States Government purposes. The Department of Energy will provide public access to these results of federally sponsored research in accordance with the DOE Public Access Plan (http://energy.gov/downloads/doe-public-access-plan).

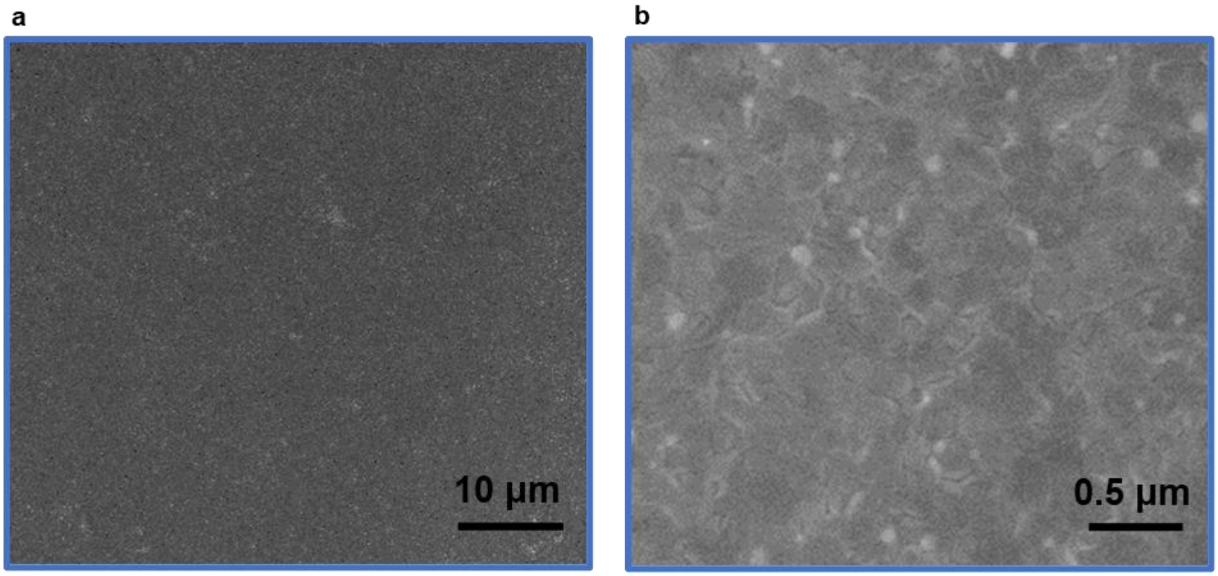

Figure S1. Scanning electron microscopy images with different field of view of the $CH_3NH_3PbI_3$ thin film used in this study.

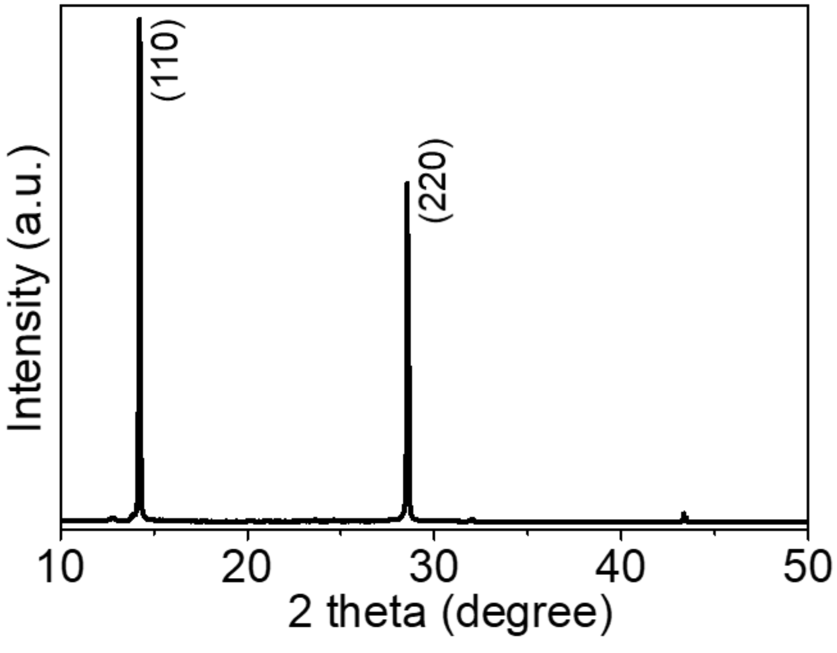

Figure S2. The X-ray diffraction patterns of the $CH_3NH_3PbI_3$ thin film used in this study, which indicate the good crystallinity of the sample.

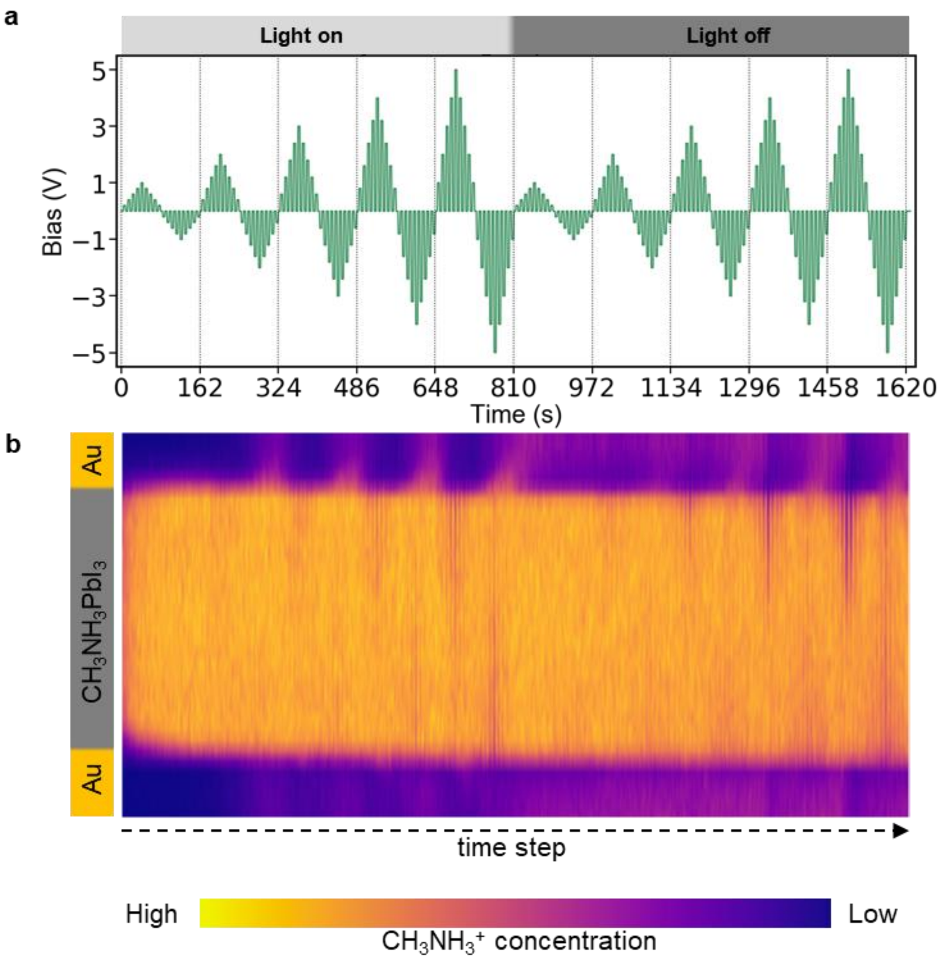

Figure S3. A tr-ToF-SIMS results showing temporal $CH_3NH_3^+$ distribution during the application of a gradually increasing pulsed stepwise electric waveform. a, a full voltage-time (V-t) curve that applied during tr-ToF-SIMS measurement; **b,** obtained tr-ToF-SIMS results of temporal $CH_3NH_3^+$ distribution. Here, the first half measurement was conducted under light-on condition and the second half measurement was conducted under light-off condition in order to study the light effect on ion migration.

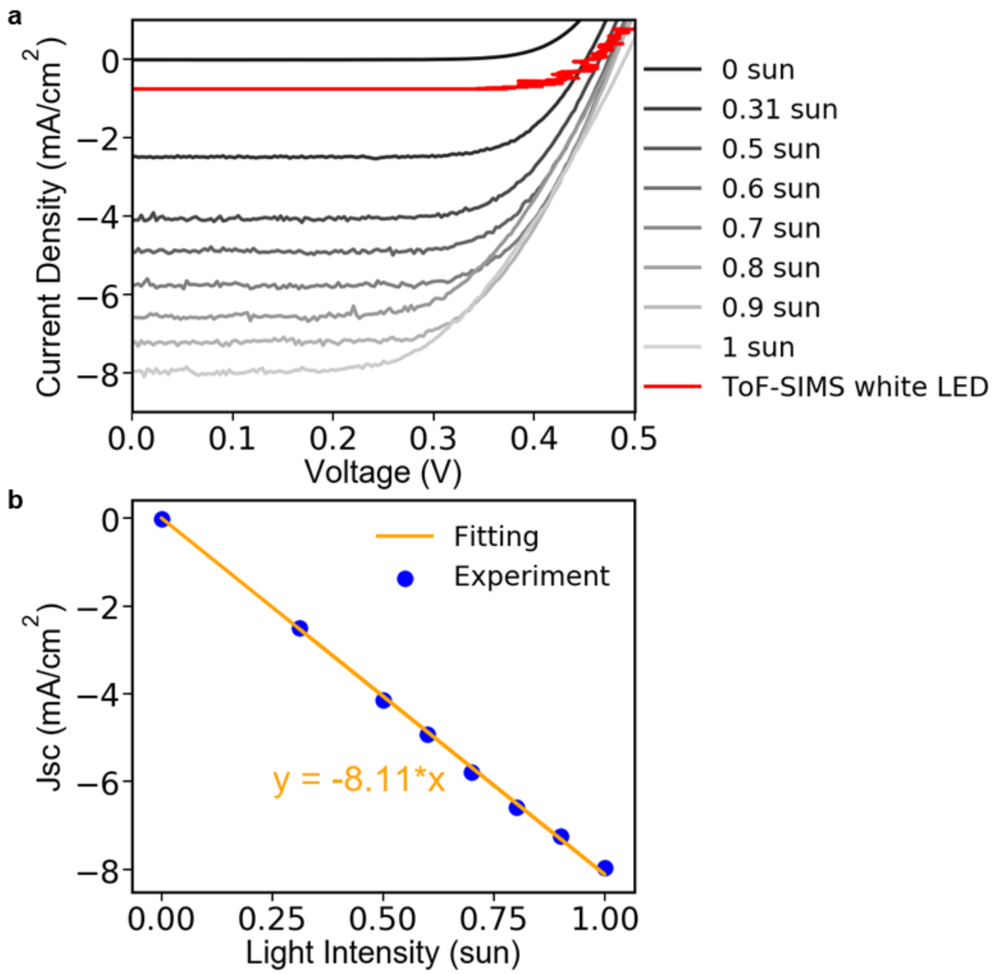

Figure S4. Characterization of the intensity of white light-emitting diode (LED) used in tr-ToF-SIMS measurement by measuring the performance of a silicon solar cell under illumination of the white LED. **a,** the current-voltage curves of the silicon solar cell under various intensity of sunlight illumination and the white LED light. **b,** the J_{sc} of the silicon solar cell as a function of the sunlight intensity, which can be linearly fitted as shown. According to this fitting result and the J_{sc} of the silicon solar cell under the white LED illumination (~0.76 mA/cm^2), we calculate the intensity of the white LED is equivalent to 0.094-sun. We note that this method only provides a rough intensity of the white LED, the calculated intensity is not accurate due to the wavelength mismatch between the white LED and sunlight

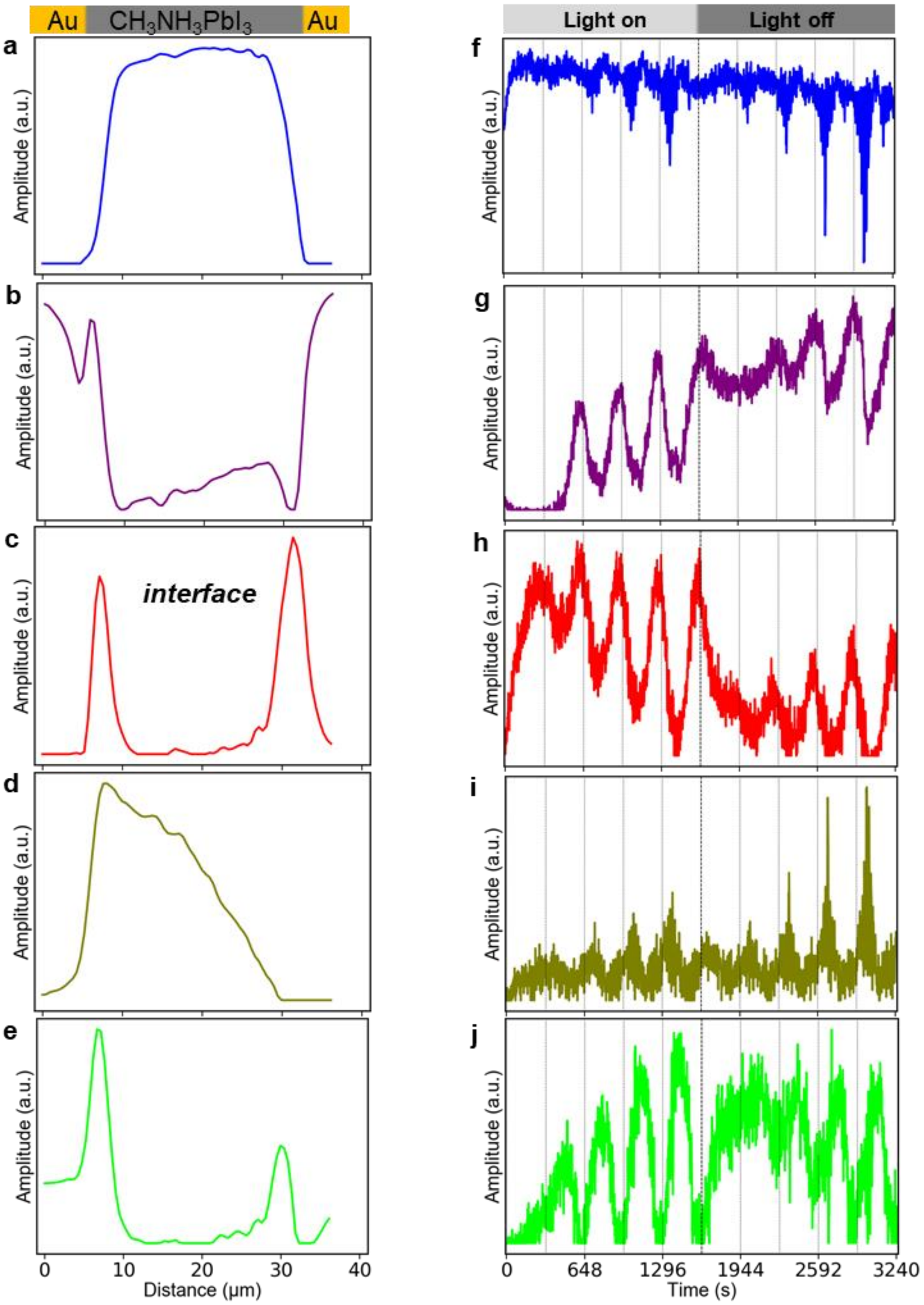

Figure S5. Non-negative matrix factorization (NMF) analysis of the tr-ToF-SIMS result in Figure S3. a-e, the tr-ToF-SIMS result was spatially isolated into five endmembers, which represent $CH_3NH_3^+$ distributed at the respective regions. The endmember in **(c)** that represents $CH_3NH_3^+$ distributed at Au/$CH_3NH_3PbI_3$ interface is what we are interested in, which was named

as endmember *interface*; **f-j,** the time evolution of the endmembers showing in **(a-e),** respectively. all evolutions are consistent with the applied electric waveform in **Figure S3a**.

Notes to Figure S5:

Five endmembers are shown as $CH_3NH_3^+$ distribution profiles (Figure S5a-e) across the lateral device, a schematic of the lateral device corresponding to the profiles is shown above the Figure S5a. Compared all endmember profiles to the schematic of the lateral device, we can find that the endmember shown in Figure S5c represent the $CH_3NH_3^+$ distributed near the device interfaces. In addition, the evolutions of each endmember with time (bias and illumination conditions vary with time) are shown in Figure S5f-j, respectively; the x-axis in Figure S5f-i corresponds to the time-step of tr-ToF-SIMS map in Figure S3b, where the bias condition is also shown in Figure S3a. These evolutions represent the $CH_3NH_3^+$ intensity change in the respective region—for example, Figure S5c represents the $CH_3NH_3^+$ distributed near the device interfaces, so the corresponding evolution in Figure S5h indicates the $CH_3NH_3^+$ intensity change near the device interfaces with time.

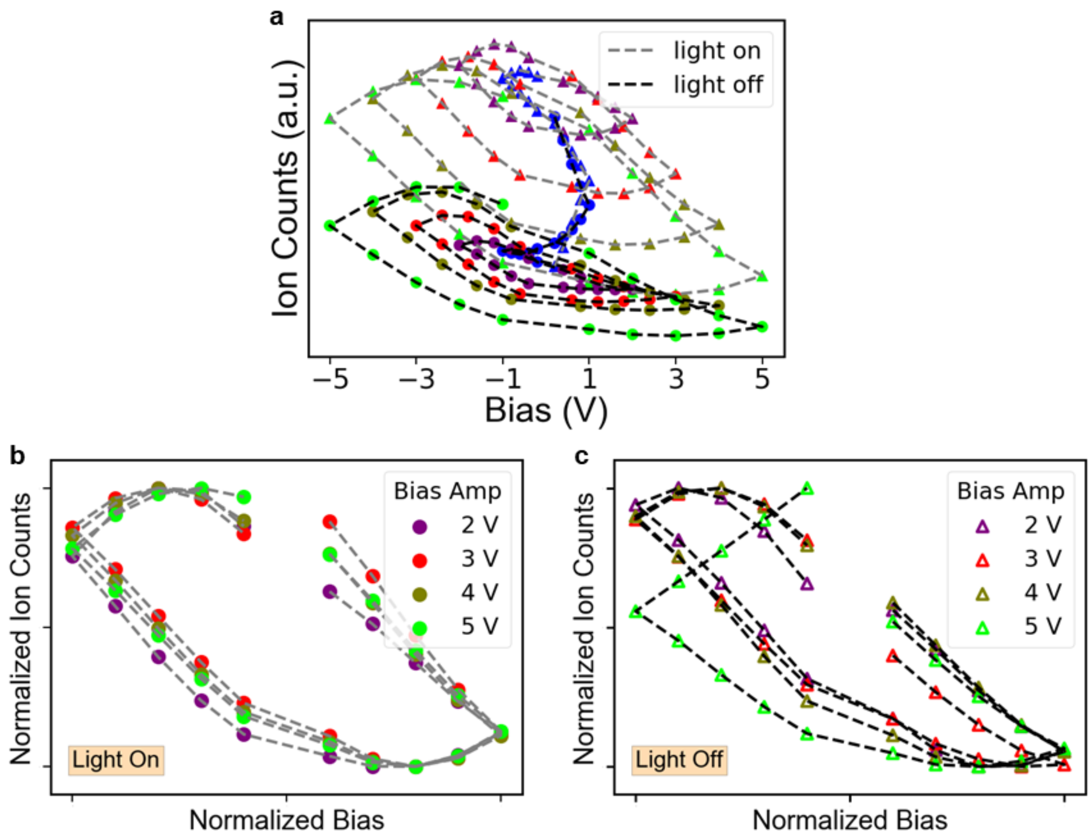

Figure S6. a, the evolution of the endmember *interface* shown in **Figure S5c** as a function of the applied electric voltage, which show hysteretic $CH_3NH_3^+$ migration; **b, c,** normalized (both ion counts (y-axis) and bias (x-axis) are normalized) loops of hysteretic $CH_3NH_3^+$ migration show in (**a**) under light-on and light-off, respectively, which show that the hysteresis loops are the same when the bias amplitude increases from 2 V to 5 V.

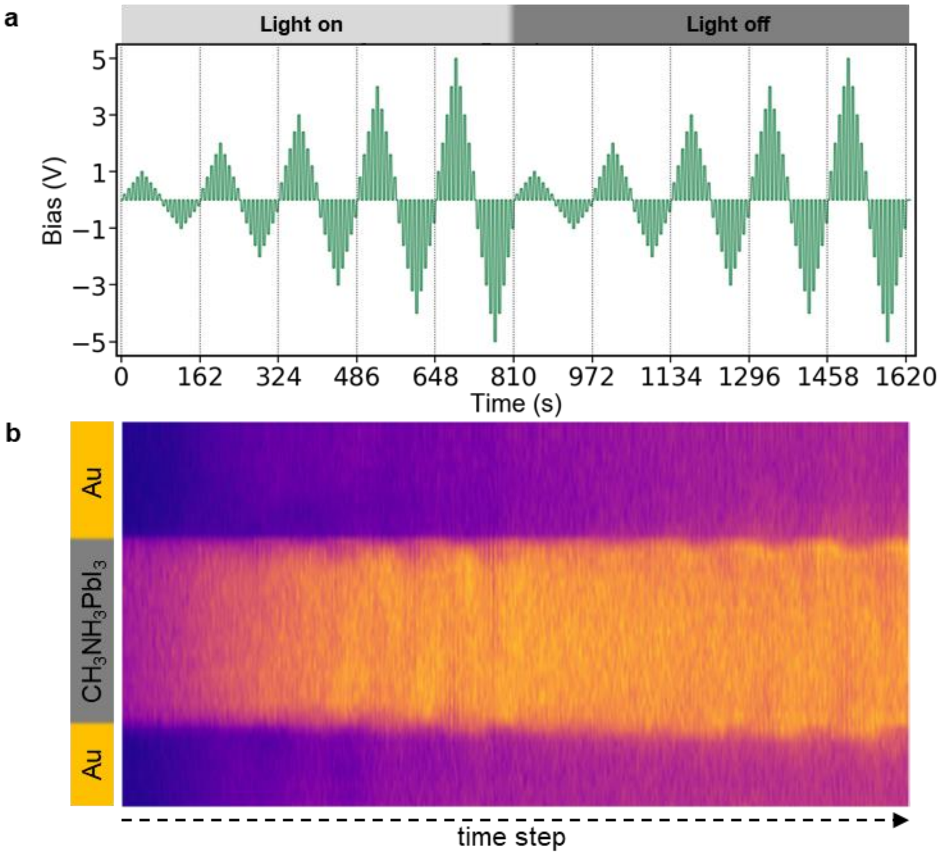

Figure S7. A tr-ToF-SIMS results showing temporal I$^-$ distribution during the application of a gradually increasing pulsed stepwise electric waveform. a, a full voltage-time (V-t) curve that applied during tr-ToF-SIMS measurement; **b,** obtained tr-ToF-SIMS results of temporal I$^-$ distribution. Here, the first half measurement was conducted under light-on condition and the second half measurement was conducted under light-off condition in order to study the light effect on ion migration.

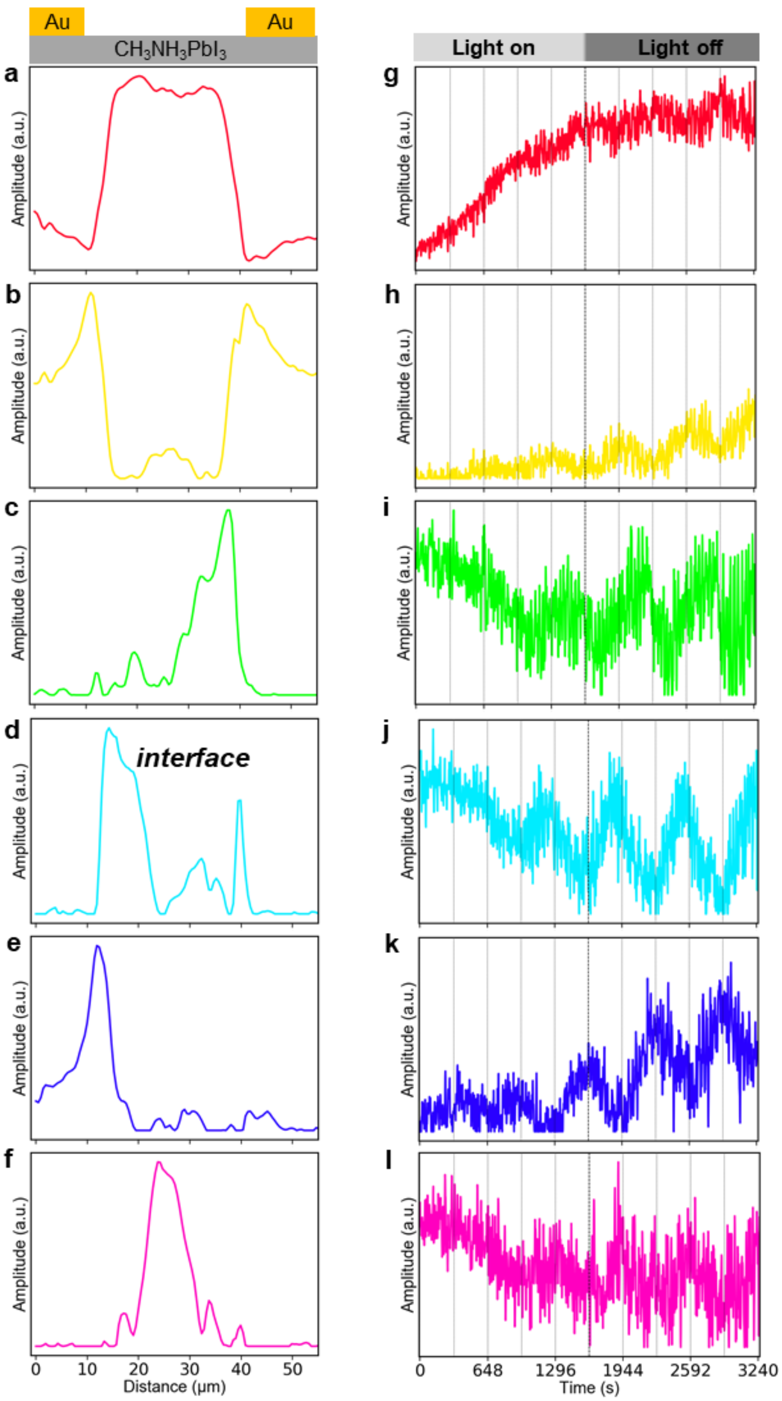

Figure S8. Non-negative matrix factorization (NMF) analysis of the tr-ToF-SIMS result in Figure S7. a-e, the tr-ToF-SIMS result was spatially isolated into six endmembers, which represent I^- distributed at the respective regions. The endmember in **(d)** that represents I^- distributed at Au/$CH_3NH_3PbI_3$ interface is what we are interested in, which was named as

endmember *interface*; **f-j,** the time evolution of the endmembers showing in **(a-e),** respectively. all evolutions are consistent with the applied electric waveform in **Figure S7a**.

Notes to Figure S8:

Six endmembers are shown as I^- distribution profiles (Figure S8a-f) across the lateral device, a schematic of the lateral device corresponding to the profiles is shown above the Figure S8a. Compared all endmember profiles to the schematic of the lateral device, we can find that the endmember shown in Figure S8d represent the I^- distributed near the device interfaces. In addition, the evolutions of each endmember with time (bias and illumination conditions vary with time) are shown in Figure S8g-l, respectively; the x-axis in Figure S8g-l corresponds to the time-step of tr-ToF-SIMS map in Figure S7b, where the bias condition is also shown in Figure S7a. These evolutions represent the I^- intensity change in the respective region—for example, Figure S8d represents the I^- distributed near the device interfaces, so the corresponding evolution in Figure S8j indicates the I^- density change near the device interfaces with time.

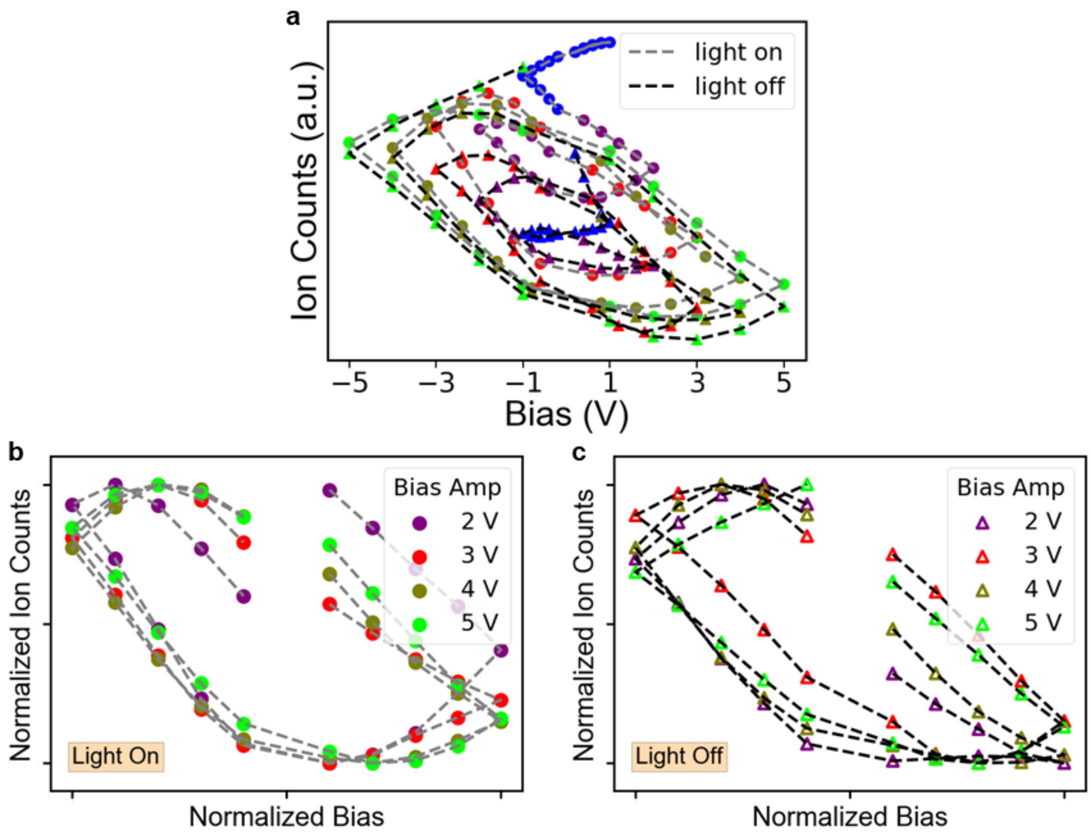

Figure S9. a, the evolution of the endmember *interface* shown in **Figure S8d** as a function of the applied electric voltage, which show hysteretic $CH_3NH_3^+$ migration; **b, c,** normalized (both ion counts (y-axis) and bias (x-axis) are normalized) loops of hysteretic $CH_3NH_3^+$ migration show in (**a**) under light-on and light-off, respectively, which show that the hysteresis loops are the same when the bias amplitude increases from 2 V to 5 V.

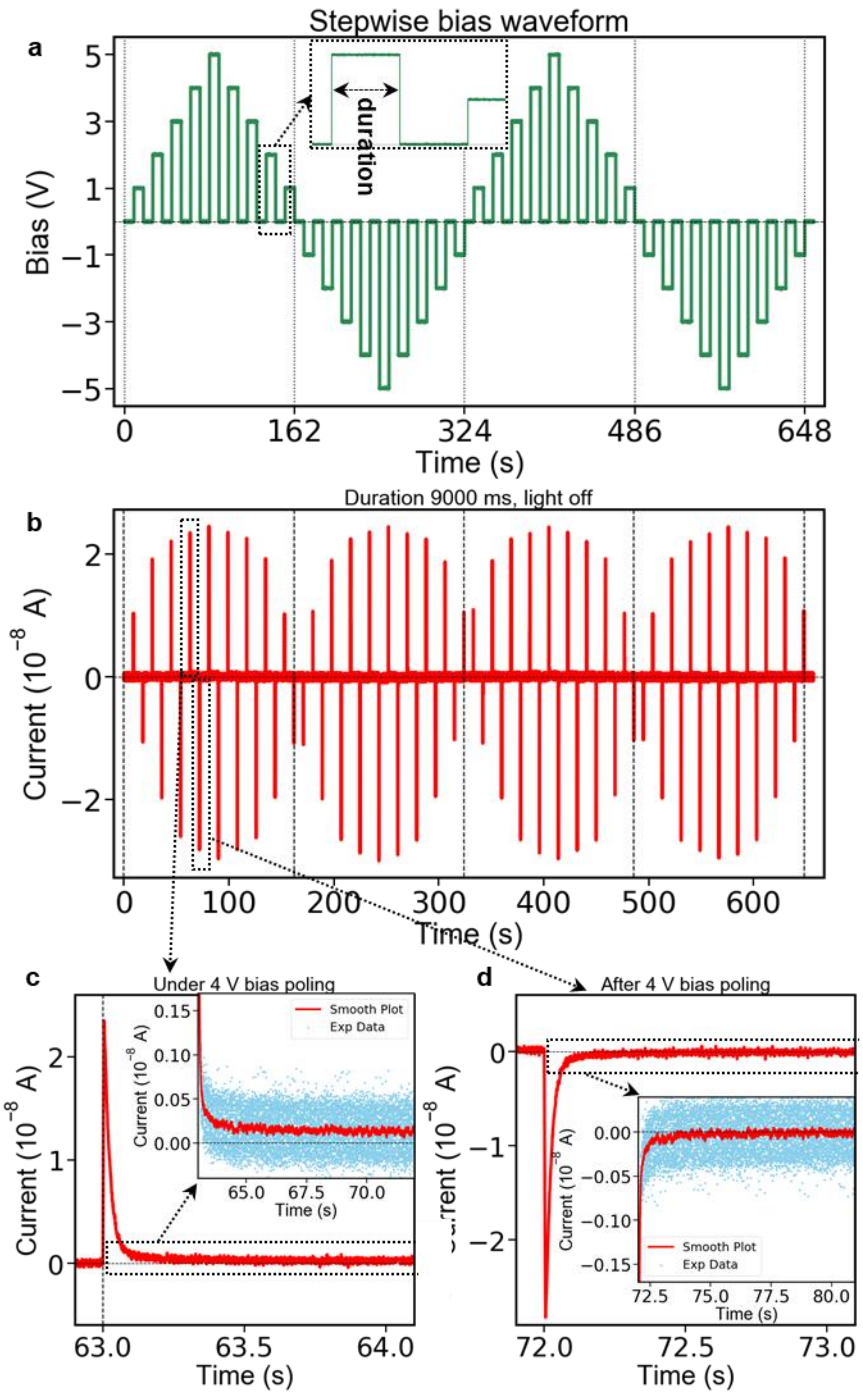

Figure S10. Electric current study by applying a pulsed stepwise waveform bias. a, the voltage-time curve (V-t) of the pulsed stepwise waveform bias applied to study the evolution of electric current, the step duration is 9 s; **b,** the obtained current-time (I-t) curve under light-off condition; **c, d,** representative enlarged figures that show on-field current decay during

application of 4 V and off-current current decay after application of 4 V, respectively; the inserts of (**c-d**) show the current evolution after the initial fast decay.

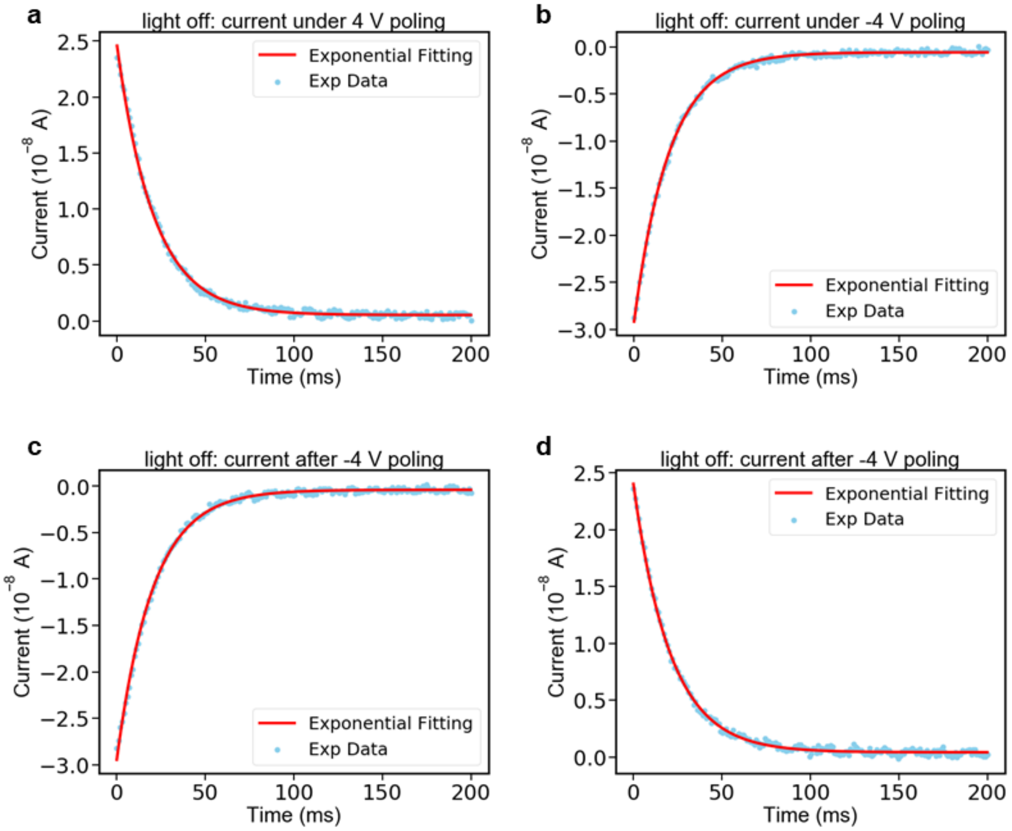

Figure S11. Representative exponential fitting of the fast decay of the currents upon application of the bias and after removal of the bias under light-off condition. a, b, exponential fitting of currents upon application of 4 V and -4 V, respectively. **c, d,** exponential fitting of currents after removal of 4 V and -4 V, respectively. All fittings are based the current during the first 200 ms.

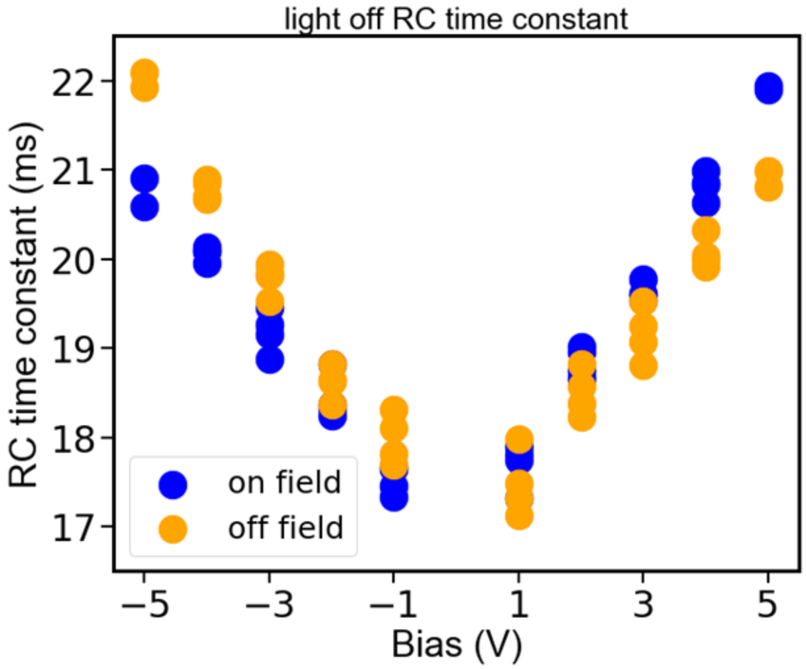

Figure S12. Light-off condition RC constant of the lateral device obtained by exponential fitting. The RC time constant is the time required for a capacitor to charge to 63% of the DC voltage charging it or the time required to discharge to 63% of its fully charged voltage, which represents the rate of charge/discharge of a capacitor through a resistor. It is shown above that the RC time constant decreases with increasing the amplitude of DC voltage, implicating the lateral device is modified by bias poling due to ion migration and/or structure distortion. We note that if the fast decay process is dominated by ion migration, we would see smaller RC time constant (suggesting faster decay) with increasing bias voltages because ions should move faster under larger bias voltages. Therefore, the trend of RC time constant also suggests that the fast decay process is not dominated by ion migration.

.

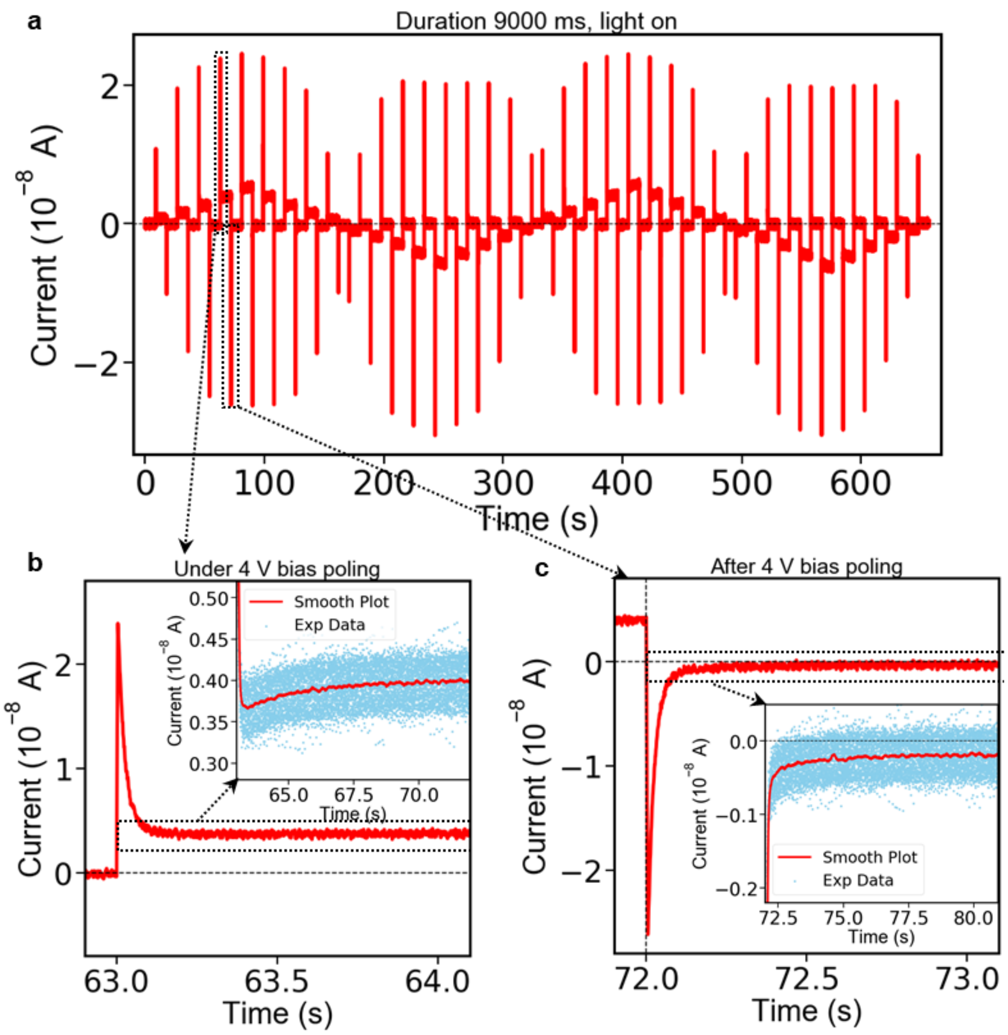

Figure S13. Electric current study by applying a pulsed stepwise waveform bias. The voltage-time curve (V-t) of the pulsed stepwise waveform bias is shown in **Figure S10a**. **a,** the obtained current-time (I-t) curve under light-on condition; **b, c,** representative enlarged figures that show on-field current decay during application of 4 V and off-current current decay after application of 4 V, respectively; the inserts of (**b-c**) highlight the current evolution after the initial fast decay.

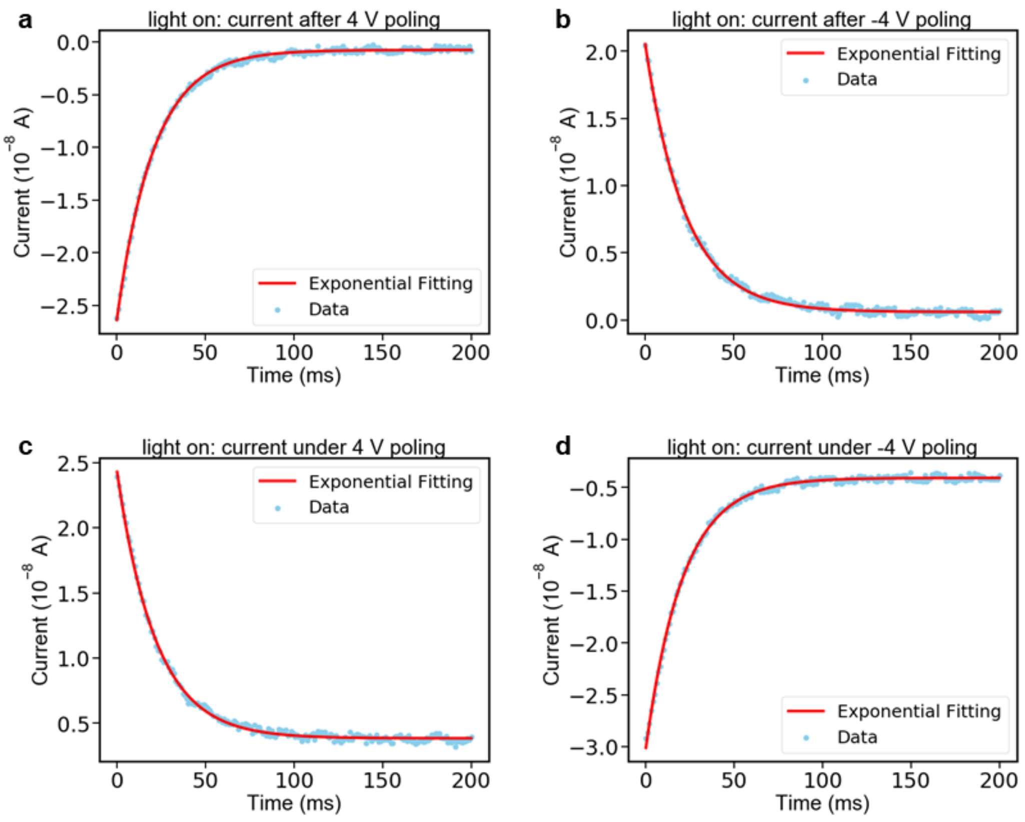

Figure S14. Representative exponential fitting of the fast decay of the currents upon application of the bias and after removal of the bias under light-on condition. a, b, exponential fitting of currents upon application of 4 V and -4 V, respectively. **c, d,** exponential fitting of currents after removal of 4 V and -4 V, respectively. All fittings are based the current during the first 200 ms.

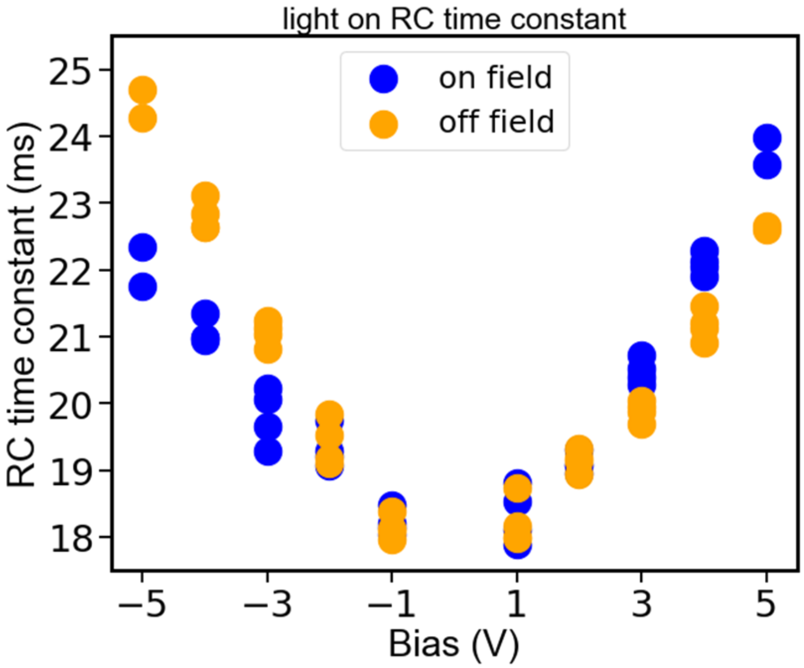

Figure S15. Light-off condition RC constant of the lateral device obtained by exponential fitting. Consistent with the light-off condition, it is shown that the RC time constant decreases with increasing the amplitude of DC voltage under light-on.

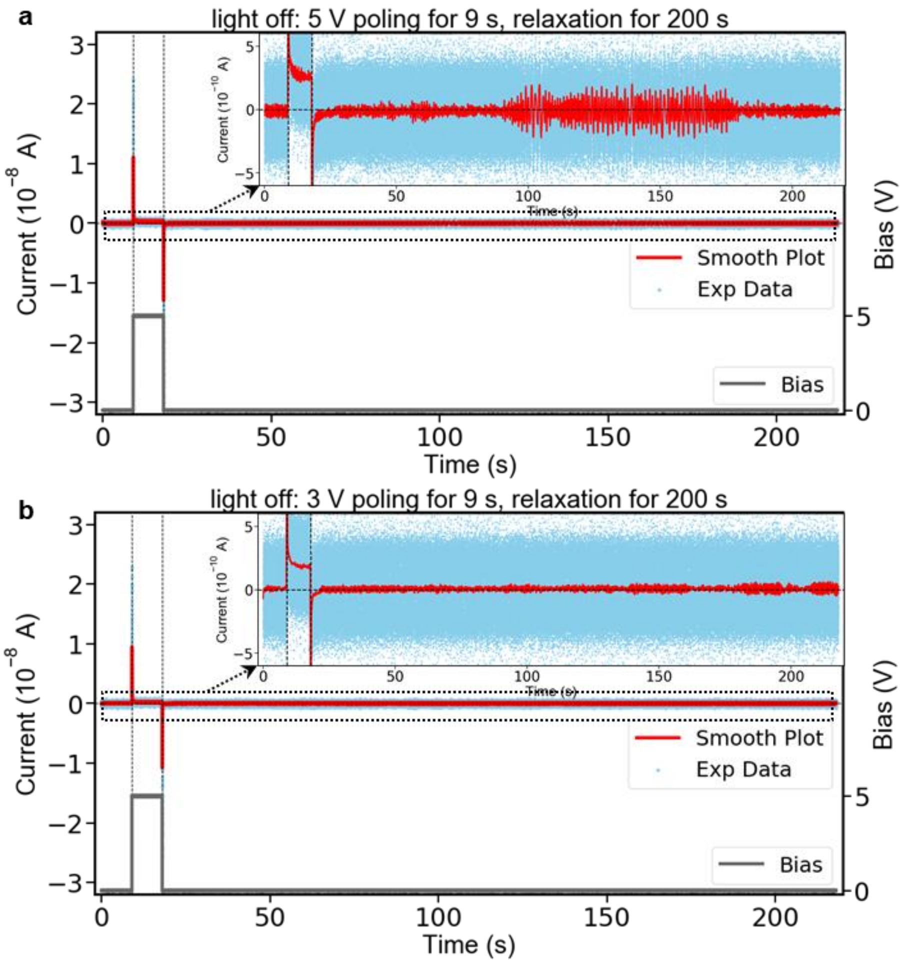

Figure S16. Current behavior under light-off. a, I-t curve by 5 V poling for 9 s and relaxing for 200 s; the insert is a zoomed-in I-t curve to show the remanent current behavior. **b,** I-t curve by 3 V poling for 9 s and relaxing for 200 s; the insert is a zoomed-in I-t curve to show the remanent current behavior.
